# Examining multi-session brief intervention for substance use in primary care: research methods of a randomized controlled trial

**DOI:** 10.1186/s13722-016-0057-6

**Published:** 2016-04-18

**Authors:** Jaclyn E. Chambers, Adam C. Brooks, Rachel Medvin, David S. Metzger, Jennifer Lauby, Carolyn M. Carpenedo, Kevin E. Favor, Kimberly C. Kirby

**Affiliations:** Treatment Research Institute, 600 Public Ledger Building, 150 S. Independence Mall West, Philadelphia, PA 19106 USA; Department of Psychiatry, University of Pennsylvania School of Medicine, 3900 Chestnut Street, Philadelphia, PA 19104 USA; Public Health Management Corporation, Centre Square East, 1500 Market St. 15th Floor, Philadelphia, PA 19102 USA; The Institute for Graduate Clinical Psychology, Widener University, One University Place, Chester, PA 19013 USA; Lincoln University, 1570 Baltimore Pike, Lincoln University, PA 19352 USA; Rowan University, 201 Mullica Hill Road, Glassboro, NJ 08028 USA

**Keywords:** Brief intervention, SBIRT, Brief treatment, Substance abuse, Primary care

## Abstract

**Background:**

Brief interventions such as Screening, a single session of Brief Intervention, and Referral to Treatment (SBIRT) have shown mixed effectiveness in primary care. However, there are indications that multi-session brief interventions may demonstrate more consistently positive outcomes, and perhaps a more intensive approach would be of benefit in addressing substance use in primary care. This study compared the effectiveness of SBIRT with a single BI session (BI/RT) to a multi-session brief-treatment intervention (BI/RT+) in primary care. We also developed easy-to-use, evidence-based materials to assist clinicians in delivering these interventions.

**Methods/design:**

This study was conducted in three Federally Qualified Healthcare Centers (FQHCs). A total of 10,935 patients were screened, and 600 individuals were recruited. The sample was primarily Black/African American (82 %) with a mean age of 40. Patients who attended a healthcare appointment were screened for substance use via the AUDIT and DAST. Patients were eligible for the study if they scored 8 or higher on the AUDIT, were using only marijuana and scored 2 or higher on the DAST, or were using other illicit drugs and scored 1 or higher on the DAST. Participants were randomly assigned to receive one-session BI/RT, or two to six sessions of brief intervention that incorporated elements of motivational enhancement therapy and cognitive-behavioral therapy (BI/RT+). Both interventions were delivered by behavioral health consultants at the FQHCs. Participants completed follow-up assessments every 3 months for 1 year. Primary outcome variables included substance use treatment sessions attended and days of substance use. Secondary outcomes included measures of health, employment, legal, and psychiatric functioning and HIV risk behaviors. Additionally, we will conduct an economic evaluation examining cost-effectiveness and will analyze outcomes from a process evaluation examining patient and provider experiences.

**Discussion:**

The ability of brief interventions to impact substance use has great potential, but research findings have been mixed. By conducting a large-scale randomized controlled trial in real-world health centers, this study will answer important questions about the effectiveness of expanded BIs for patients who screen positive for risky substance use in primary care.

*Trial registration* NCT01751672

## Background

Most Americans with diagnosable substance use disorders (SUDs) never receive treatment. The 2012 National Survey on Drug Use and Health (NSDUH) estimated that 22.2 million individuals meet criteria for a DSM-IV alcohol or drug use disorder. Prevalence estimates for alcohol use disorder alone are at 14.9 million; 4.5 million individuals meet criteria for a diagnosable drug use disorder, while 2.8 million individuals meet criteria for a combined alcohol and drug use diagnosis [1]. It is estimated that 90 % of this population do not recognize the extent of their problem and never seek treatment [2]. The specialty care system is not prepared or always appropriate for this enormous population that requires intervention for substance use [2]. Many individuals on the more mild end of the SUD spectrum would feel out of place in community-based outpatient programs, and so do not attend. Furthermore, patients who are subthreshold for a substance use disorder diagnosis and are simply using drugs or alcohol at a risky level may benefit from being advised to cut back. Since many patients with substance related issues may not seek specialty care but do seek primary care because they are dealing with numerous medical conditions (related to and complicated by their substance use) [3–5], screening and intervening for risky substance use in primary care makes intuitive sense. Screening, Brief Intervention, and Referral to Treatment (SBIRT) is an approach in which all patients in a medical setting are briefly screened for risky substance use; patients who screen positive are provided with a brief intervention (BI) that is intended to direct them to monitor and reduce substance use, and patients with moderate to severe substance use are referred to treatment (RT) and specialty care if necessary [6].

SBIRT studies have typically focused on either alcohol use or drug use, and few have examined both substances. BIs delivered in primary care for risky alcohol use have a robust history of mostly positive results. Early trials demonstrated moderate-to-high efficacy of BIs when applied to hazardous drinking in the primary care setting [7]. Bien et al. [7] conducted a review of 32 controlled trials of BIs for alcohol abuse and determined that single well-executed conversations are adequate to alter mild to moderate alcohol abuse patterns. Other studies have demonstrated little difference in outcomes for patients treated with single session interventions and patients treated with more extensive and traditional treatments [8–12]. A recent review and meta-analysis of 23 studies has shown alcohol BIs in primary care are indeed related to self-reported drinking reductions, but result in few other health or service utilization outcome differences; effects of intervention were stronger in studies where patients received multiple BI contacts in primary care [13]. Furthermore, it may be that the BI executed with patients with alcohol problems is largely impacting risky drinkers but not impacting more severe drinkers [13], as a recent meta-analyses has demonstrated that the provision of SBIRT does not seem to result in greater engagement with specialty treatment [14].

Given that SBIRT may be more effective when delivered with multiple BI contacts and the lack of evidence that the referral portion of the intervention is effective, questions remain about the impact of implementing the approach in primary care. Recent effectiveness trials and implementation studies found that SBIRT was not significantly more effective than physician advice interventions and was challenging to implement [15, 16]. One study tested three interventions for patients in primary care who screened positive for hazardous drinking—an informational leaflet, 5 min of brief advice, and 20 min of brief counseling—and found no significant differences in participants’ AUDIT scores among the three conditions at 6 and 12 months [15]. Another study aimed to implement a program wherein general practitioners provided personal feedback and support for patients with risky alcohol use, and found that the program did not significantly increase the rate of SBI being delivered [16]. These studies concluded that alcohol-targeting BIs in the primary care setting employing standard or typical implementation strategies may be difficult to enact and fail to show effectiveness at reducing hazardous or harmful drinking. Finally, concerns about BI have been raised regarding long-term efficacy and program implementation [17, 18].

Another open question regarding the use of brief intervention in primary care concerns how to best apply the approach to illicit drug use. The use of SBIRT to address illicit drug use has not been as successful as with alcohol. Early studies indicated that randomizing patients to BIs for cocaine, heroin, and prescription drugs did result in drug use reductions [19, 20] and improved engagement in specialty care [19]. The BIs in these early studies were defined as a single session with a primary care peer educator for cocaine and heroin users in one study [19], and two sessions of motivational interviewing and individualized written feedback provided by a psychologist for patients who had been identified as having problematic prescription drug use in another study [20]. The World Health Organization conducted a four-nation randomized controlled trial (RCT) of SBIRT in which each nation developed its own culturally appropriate single-session brief intervention using the FRAMES model. This study demonstrated that brief intervention did result in 3-month self-reported reductions of cannabis, cocaine, and heroin use relative to an assessment-only control, although these effects were not demonstrated in the U.S. [21]. An uncontrolled SAMHSA implementation effort of SBIRT in six health systems (screening approximately 460,000 patients) found significant reductions of illicit drug use at 6 months and self-reported improvement on functional domains including general health and mental health [22]. The SAMHSA SBIRT implementation involved progressive levels of single-session brief intervention and referral to treatment depending on substance use severity.

However, the most recent well-designed studies of BI for illicit drug use in primary care have demonstrated no efficacy on drug use, treatment entry, or service utilization [23, 24]. One study found that neither a single-session BI conducted by a health educator nor a single-session BI with a later booster session conducted by master’s-level counselors significantly reduced days of substance use as compared to a control group [23]. Another study found that a one-time brief intervention using motivational interviewing had no effect on drug use in patients as compared with usual care [24]. These rigorous studies included screening only control groups, biological verification of use, and excellent follow-up rates. The poor outcomes of BIs for illicit drug use in primary care are well summarized in a recent review that also reiterates the need to rethink what might be effective for illicit drug use [25].

While brief interventions have had more promising results for alcohol use than for illicit drug use, we hypothesized that the lack of efficacy for illicit drug use and more severe alcohol use might be explained by intensity and frequency of intervention. Perhaps, similar to the conclusions drawn by Jonas et al. [13], multiple linked BIs for illicit drug use and more severe alcohol use would be more effective than a single session of SBIRT. We hypothesized that severe alcohol users and illicit drug users could potentially respond to a more expansive intervention that incorporates elements of brief treatment. For instance, four sessions of expanded brief intervention approaches such as Motivational Enhancement Treatment (MET; [26]) have been demonstrated to be as effective as 12 sessions of cognitive-behavioral therapy (CBT) or 12-step facilitation treatment for the treatment of alcohol [27]. Motivational Interviewing (MI) and MET have been demonstrated to be effective in reducing alcohol and drug use and improving engagement with specialty care [28–30]. Similarly, brief cognitive-behavioral approaches (1–6 sessions) have been demonstrated to be effective in the treatment of amphetamines, cannabis, and cocaine [31–33] (on some measures but not all, and again, more sessions seem to result in better outcomes). MI strategies have been successfully combined with cognitive-behavioral approaches for the brief treatment of alcohol in primary care settings [34], as well as for treating cannabis [35]. Implementing a multi-session brief intervention could be a promising approach to treating substance abuse in primary care that warrants further investigation, particularly for patients with mild to moderate SUDs, or for patients using only alcohol or marijuana, which may be perceived as social/recreational and less harmful.

We know that efforts to integrate primary care and addiction treatment have resulted in improved substance and medical outcomes [36–38]. Various office-based brief treatments, if repackaged for a primary care setting, could potentially be effective for severe alcohol and illicit drug use. Furthermore, such interventions may increase compliance as it has been demonstrated that patients feel less stigma discussing substance use with a medical professional than seeking specialty care [39]. Finally, for patients who do indeed require additional SUD treatment through a referral to specialty care, the expanded number of brief intervention contacts in primary care could conceivably result in improved follow-through in community treatment engagement, with increased attention for resolving ambivalence and negotiating obstacles to treatment.

Implementation barriers are also important to consider when examining SBIRT in a primary care setting. When patients meet criteria for more than mild substance use, they are intended to be referred to treatment and ongoing monitoring [6]. This step is often problematic, as primary care providers may be unfamiliar with the specialty care system or may find it unsatisfactory for addressing patients’ problems [40]. Furthermore, patients who do not meet criteria for dependence or who are only using substances they perceive as recreational (alcohol or cannabis) may feel as though they do not fit into the specialty care system, which has a high concentration of judicially mandated patients [39]. In these cases, expanding a single-session brief intervention into a spaced multi-session intervention combined with ongoing monitoring may be adequate to address moderate illicit drug problems. However, simply because expanded intervention might be effective does not mean that it will be perceived as desirable or valuable for clinicians to spend time on, or that it will be deployed given the various pressures encountered in Federally Qualified Healthcare Centers (FQHCs). Additionally, referral to treatment may still be warranted in some cases depending on the needs of the patient. While these issues and considerations are relevant to all primary care providers, they are particularly important for FQHCs. FQHCs are required to offer referrals to substance use providers, and many continue to face challenges when determining how to best implement SBIRT procedures [41].

### Present study

Our team is testing the efficacy of expanded brief interventions, with referral to treatment as needed, in FQHCs among patients who have risky alcohol and/or drug use. We selected FQHCs for the study sites because they serve a diverse underserved population, and behavioral health services are already embedded. With an interdisciplinary, team-based approach, FQHCs address the full spectrum of their patients’ needs. In 2010, FQHCs served approximately 15 % of the total U.S. uninsured population, 15.5 % of Medicaid enrollees, and 6.4 % of the total U.S. population, representing an estimated 20 million patients [42]. Of those patients served, 62 % were members of a racial or ethnic minority, 93 % lived at or below 200 % of the federal poverty level, and 38 % were uninsured [42]. FQHCs are estimated to expand coverage to 50 million people by 2019 [43]. Additionally, the FQHCs’ ability to derive revenue from multiple payers bolsters the sustainability of the interventions.

This study will compare the effectiveness of an enhanced, multi-session brief intervention (akin to brief treatment) and ongoing monitoring (BI/RT+) to SBIRT with a single BI session (BI/RT) to improve treatment engagement and substance use outcomes, as well as other psychosocial outcomes, amongst substance users. We conceptualized this project as both an efficacy trial and a demonstration/implementation project, as we systematically developed behavioral health intervention tools to support fidelity. We will also examine the implementation processes and economic impact and sustainability of our experimental intervention. Our mixed efficacy/effectiveness study will also add to an understanding of whether these expanded interventions fit into the clinical flow of FQHCs, and whether they are cost-effective.

## Methods/design

This study implemented a high fidelity SBIRT protocol in three primary care clinics in urban Philadelphia and trained six behavioral health consultants (BHCs) in an expanded brief intervention protocol (BI/RT+). The aims of this study are as follows:

**Aim 1:** To conduct a randomized controlled trial to address the following ***Primary Hypotheses***:

### **H1**

Patients assigned to BI/RT+ will attend more substance intervention and specialty treatment sessions outside of the FQHC over the 12-month follow-up than patients assigned to BI/RT.

### **H2**

Patients assigned to BI/RT+ will demonstrate larger reductions in drug and alcohol use by point prevalence urine samples and by reported days using over the 12-month follow-up compared to patients assigned to BI/RT.

### **H3**

BI/RT+ will have positive net social benefits relative to BI/RT alone.

### **Secondary Hypotheses (H4)**

Patients assigned to BI/RT+ will demonstrate improved medical, employment, legal, and psychiatric functioning, as well as reduced HIV risk over the 12-month follow-up compared to patients assigned to BI/RT.

**Aim 2:** To determine whether BI/RT and BI/RT+ are sustainable in primary care clinics.

**Aim 3:** To conduct a process evaluation of BI/RT+ to assess implementation barriers and workforce attitudinal shifts.

### Subject population and setting

A total of 10,935 patients were screened, and 600 eligible individuals were recruited to participate. All participants were patients at three primary care clinics in the Philadelphia metro area. The three clinics in this study are all FQHCs serving traditionally underserved populations, including many low-income and uninsured patients. In addition to providing primary care, the FQHCs offer supportive services such as case management, health education, and prevention using an interdisciplinary approach. The study primarily included adults over the age of 21, but older adolescents (i.e., 18–21 years) were not excluded. All three of the FQHCs are located in neighborhoods of Philadelphia that have greater minority representation than is found for the city as a whole. Our sample was approximately 82 % Black, 7 % White, and 12 % other races. About 8 % were Hispanic and approximately 45 % of our participants were women; the average age was 40. About 30 % were employed either full-time or part-time, 66 % were never married, 11 % were married or cohabitating, and 75 % had at least a high school diploma or equivalent. There was a fairly even split of primary substance use; 35 % of participants primarily used alcohol, 38 % marijuana, and 27 % other substances.

### Screening and recruitment

Patients who attended a healthcare appointment at the three participating clinics were asked to complete a brief screener regarding their alcohol and drug use. Results of the screening were immediately available for the primary care practitioner to review in the privacy of the exam room during the patient visit. The practitioner was responsible for (a) reviewing the participants’ screener responses, (b) having a brief conversation with them about their responses, and if appropriate, (c) inviting them to speak with a BHC. If the patient agreed, the BHC engaged in 5–10 min of brief intervention using motivational interviewing strategies to open a non-threatening discussion about the substance use and conducted more in-depth screening to determine the severity of the patient’s substance use. If both the practitioner and the BHC determined that the patient was appropriate for the study, the BHC informed the patient about the study and introduced the patient to a research assistant who invited participation. Patients who agreed to be in the study then completed an informed consent process. Of the 10,935 patients who completed the initial self-report screener, 4232 were flagged for potentially risky substance use. BHCs were able to conduct in-depth screening with 2011 of these patients, and 871 were found to be eligible for the study. There were 2221 patients who were flagged on their initial screener but did not receive a follow-up screening. The majority of these patients were not given an in-depth screening because they were already in substance use treatment (1060 cases). In the remaining cases, either the medical provider did not refer the patient to a BHC (451 cases) or the BHC was unavailable for a consultation (111 cases).

### Eligibility measures

Patients’ eligibility for the study was determined by their responses on the Alcohol Use Disorders Identification Test (AUDIT; [44]) and the Drug Abuse Screening Test (DAST-10; [45]). Both the AUDIT and DAST-10 have been widely validated on different patient populations and have been found to have good sensitivity and specificity for identifying patients at risk for misuse of alcohol or other drugs [44, 46–48].

### Inclusion and exclusion criteria

*Inclusion Criteria:* (a) patient was 18 years or older and (b) patient had AUDIT/DAST-10 screening scores that indicated at least mild problem severity for drug or alcohol use (see Table 1).

**Table 1:** Screening criteria for inclusion of participants with at least mild problem severity

*Drug*	*Criterion*
Alcohol	AUDIT ≥ 8
Marijuana	> weekly use or DAST score ≥ 2
Other illicit drugs	DAST score ≥ 1

*Exclusion Criteria:* (a) the medical practitioner or BHC determination that medical or psychiatric complications exist that would contraindicate research participation, or because after questioning the patient it appeared that the substance use was too mild to warrant further intervention, (b) the patient reported plans to leave the Philadelphia greater metropolitan area within the next 12 months and thus would not be available for follow-up assessments, or (c) the patient was unable to provide valid informed consent.

These inclusion and exclusion criteria were chosen to make the study as inclusive and representative of the clinic population as possible. Our eligibility criteria allowed us to approach every participant 18 years or older that has a screening score that indicates risky substance use, as even those with mild problem severity may benefit from the interventions, while still also allowing patients with scores indicating a more significant problem to be included.

### Intervention procedures

All participants were screened for risky substance use prior to being invited to enroll in the study. Eligible participants consenting to the study were assigned to BI/RT or BI/RT+ via a block randomization procedure. Half of the patients identified with risky substance use received BI/RT and half received BI/RT+. Research assistants were responsible for assigning participants to conditions using a website programmed with the allocation sequence created by the study’s statistician. Participants, BHCs delivering the intervention, and research assistants were not blinded to group assignment.

In both conditions, all counseling sessions were digitally audio-recorded and securely stored in order to complete supervision and intervention integrity analyses. Throughout the trial, a clinical supervisor and expert in SBIRT reviewed 1 to 2 randomly selected sessions for each BHC each month and provided at least bi-weekly supervision with BHCs to (a) review the BI/RT or BI/RT+ content covered in meetings, (b) reinforce the BHCs’ adherence to the protocol, (c) discuss any protocol breaks that occurred in prior patient meetings, and (d) address any therapeutically relevant issues that might be occurring (e.g., new problems, ethical issues).

#### BI/RT

Participants in the BI/RT condition attended one 20–40 min audio-taped brief intervention session with the BHC that was intended to approximate standard SBIRT delivered by a BHC in a FQHC setting. This session occurred at the primary care site on the same day that the participant enrolled into the study. The BHC employed motivational interviewing techniques (MI; [49]) to explore the patients’ perceptions of their substance use. Exploring a referral to treatment was a standard component that was provided as part of the BI/RT intervention; the type of referral that was made depended on the severity of the patient’s substance use. Patients with scores indicating they have “mild” severity (occasional risky/illicit use) were encouraged to curtail illicit drug use and reduce alcohol use. Patients with scores in the “moderate” range were referred to brief treatment resources and self-help resources in the community. Patients whose scores on the screening instruments placed them in the “severe” range were referred to specialty care in the community. Level of specialty care referral (e.g., detoxification, residential, intensive outpatient) was determined based on degree of physical dependence symptoms that the patient disclosed.

The BHC followed up the initial brief intervention with a telephone call 3–4 weeks after the session. This call was intended to review the patient’s efforts to make reductions in substance use, as well as efforts to engage in 12-step recovery meetings or to follow up on treatment referrals. If necessary, the BHC offered alternate treatment referrals and/or resources.

#### BI/RT+

Participants in the BI/RT+ condition attended 2–6 (20–40 min each) audio-taped sessions that were intended to approximate the provision of severity-appropriate expanded brief intervention. The first session occurred at the primary care site on the same day that the participant enrolled into the study, and subsequent sessions occurred at the primary care site within the following 12 weeks. Similar to the BI/RT condition, the BHC employed MI techniques to explore the patient’s perspective on their substance use. However, the BHC modeled their motivational techniques after the Motivational Enhancement Therapy (MET) approach employed in Project Match [26]. This approach spreads brief intervention over sessions, allowing the BHC more time to negotiate a change plan with the patient. In addition, the BHC had numerous tools, described in more detail below, that they could employ over these multiple brief intervention sessions to assist patients in achieving their goals. Just as in the BI/RT condition, exploring a referral to treatment was a standard component of BI/RT+. Patients with scores in the “mild” and “moderate” severity range received MET geared towards helping them set a reduction or abstinence goal and a strategy for following up on this goal. “Mild” and “moderate” severity patients were generally not referred out to community counseling resources, rather they were offered up to six sessions of brief intervention that incorporated cognitive-behavioral strategies or 12-Step facilitation strategies to assist them in pursuing their reduction or abstinence goals. Patients who screened in the “severe” problem range were typically referred out to specialty care. However, the BHC was instructed to utilize repeated sessions of brief intervention to resolve treatment entry barriers with patients. Furthermore, after referred patients connected with specialty care, the BHC could offer 1–2 interventions to process patient reaction to the treatment experience and augment motivation to remain in specialty care.

BHCs provided ongoing telephone monitoring to patients assigned to BI/RT+ using RecoveryTrack^®^. RecoveryTrack^®^ is a patient interview conducted by the BHC over the phone and includes a web-based monitoring instrument that allows a treatment system to tailor a selection of outcome items to follow throughout the course of substance abuse treatment. The BHCs used the information they collected on RecoveryTrack^®^ to monitor the BI/RT+ patients and to determine if they needed any additional information, services, or treatment referrals.

### Intervention materials

Our team developed a flexible brief treatment toolkit that a behavioral health clinician can use to deliver standard SBIRT, various MET and CBT interventions, as well as basic 12-Step Facilitation and SUD treatment psychoeducation. It comprises a menu of 35 loosely linked interventions adapted for primary care; each intervention can be covered in 5–15 min, and includes patient takeaways so that concepts can be revisited out of the office. Much of the content of the “toolkit” has been presented neutrally around the concept of breaking habits, rather than focusing only on substance use, so the materials are useful for other purposes. The BHC introduces the toolkit cards during a counseling session, and encourages the patient to use the cards as educational materials and reference resources after leaving the session. Additionally, we designed a graphic novel as a supplemental component of the toolkit. The graphic novel introduces patients to two characters engaging in change related to substance use, and it provides patients with health information and activities to promote behavioral change.

### Patient follow-up procedures and outcome measures

Patients in both the BI/RT and BI/RT+ conditions were asked to complete follow-up assessments every 3 months following baseline for 1 year (i.e., at 3, 6, 9, and 12 months post-recruitment). Assessments took place in person, generally at the health center where the patient was recruited.

Table 2 provides a summary of each of the variables involved in the trial and indicates the relevant purpose of the variable, the instrument used to assess that variable, and the assessment points where the instrument was delivered.

**Table 2:** Outcome variables and assessment points

Variable	Purpose	Instrument	Assessment points
Drug and alcohol problems	Screen, randomize	AUDIT, DAST-10	Screening
Intervention and treatment engagement	Hypothesis 1	NSMOS, patient charts	Baseline, 3-, 6-, 9-, 12-months
Drug use and severity	Hypothesis 2	ASI, urinalysis, MINI	Baseline, 3-, 6-, 9-, 12-months
Medical and psychosocial functioning and HIV risk	Hypothesis 3	ASI, HIV-RA, chronic conditions Questionnaire, healthy days	Baseline, 3-, 6-, 9-, 12-months
Readiness to change and personal stress	Covariates	Personal rules, PSS-4	Baseline, 3-, 6-, 9-, 12-months
Patient satisfaction	Evaluation	Client satisfaction form	3-months

#### Outcome measures

Primary endpoints of substance use will be measured using an abbreviated version of the *Addiction Severity Index-6th Edition (ASI6)* [50, 51] and urinalyses. The ASI is a multi-dimensional interview used to measure problem severity of substance use, health, and social problems. We used an abbreviated version of the ASI6 that takes approximately 20 min to complete.

*Urinalysis* Urine analyses were conducted using an 8-panel screen that covers the most commonly abused illicit drugs, including marijuana, cocaine, amphetamine, methamphetamine, morphine/opiates, benzodiazepines, barbiturates, and PCP.

*Non*-*Study Medical and Other Services (NSMOS)* The NSMOS was specifically developed for this study and records the number of additional community and specialty care treatment services that patients received for medical, psychiatric, and drug and alcohol needs. The NSMOS also assesses whether the patient received these services in outpatient or acute care settings.

*Severity past year: MINI Plus 5.0.0* The MINI is a short, structured diagnostic interview for DSM-IV and ICD-10 psychiatric disorders [52, 53].

*Chronic Conditions Questionnaire* The Chronic Conditions Questionnaire assesses how well patients manage chronic diseases or conditions.

*Healthy Days Symptoms* The Healthy Days Symptoms measures the number of days over the past month that a patient was affected by symptoms such as pain and anxiety, as well as the number of healthy days.

*Perceived Stress Scale 4*-*Item Version (PSS-4)* The PSS is a global measure of the perception of stress, and has been shown to be correlated with quitting smoking and other measures of health behaviors [54].

*Personal Rulers* The Personal Rulers measure a patient’s perception of importance, readiness, and confidence in reducing or quitting their drug and/or alcohol use.

*HIV Risk Assessment (HIV-RA)* The HIV Risk Assessment provides a brief self-report measure of HIV testing history and sexual risk.

*Client Satisfaction* A Client Satisfaction Form was administered to participants at the 3-month follow-up to assess participant satisfaction and perceived usefulness of the interventions.

### Counselor fidelity measures

As described earlier, all counseling sessions in both conditions were audio recorded, and a random selection of tapes was reviewed by a clinical supervisor to monitor fidelity. We trained research assistants to code a randomly selected 20 % of recordings for intervention integrity using an SBIRT checklist and key BI/RT or BI/RT+ component items from the Yale Adherence and Competence Scale (YACS; [55]). The YACS is a scale for the assessment of clinician adherence to protocol and competence in delivering substance abuse treatment. The SBIRT checklist contained a list of all BI/RT and BI/RT+ activities. For 25 % of the selected recordings, a second research assistant independently completed the integrity instruments for reliability purposes.

### Process evaluation and economic evaluation

In addition to the patient-level outcomes that we are assessing in the randomized controlled trial, we will analyze results from a process evaluation and will conduct an economic evaluation to further examine the impact of the BI/RT and BI/RT+ interventions.

#### Process evaluation

The data collected for the process evaluation focuses on quantitative and qualitative data from both providers and patients that will help in the interpretation of study results. The process data includes information about training of staff, the level of exposure of patients to the proposed intervention and the intensity of services received, and experiences of providers and patients and their satisfaction with the services being delivered. Data specifying the intensity of the enhanced brief intervention (BI/RT+) will be correlated with outcome measures of interest (e.g., reductions in substance use). Other data, such as the experiences of providers implementing the BI/RT and BI/RT+ protocols, will be useful for understanding the replicability in other primary care settings and the perceived burden of obtaining the changes observed relative to perceived and measured benefits. Data was primarily collected through semi-structured interviews with providers and patients, along with supporting quantitative data (e.g., length of implementation period, number of providers trained and frequency of trainings, number of BHC visits patients in the BI/RT+ condition received).

#### Economic evaluation

The economic evaluation has two primary components: (1) a societal-level cost-effectiveness evaluation and (2) a sustainability evaluation.

From a societal perspective, we hypothesize that BI/RT+ will have positive net social benefits relative to BI/RT alone. Net social benefits will be estimated as the difference in the additional benefits in dollar terms for BI/RT+ patients relative to BI/RT patients and additional costs for BI/RT+ patients relative to BI/RT. Benefits assessed will include economic benefits from crime avoided, enhanced job productivity, and downstream medical cost offsets. Costs assessed will include the costs of the intervention, such as fixed clinic costs, time and resources expended by clinic personnel delivering the intervention, and personal costs incurred by participants receiving the intervention (e.g., transportation expenses).

In addition to performing a societal-level economic evaluation, we will also develop a sustainability model for primary care clinics to help inform a decision regarding adoption of BI/RT or BI/RT+ in the future. Inputs for the model will consist of fixed costs for the intervention, such as training and space allocation, as well as variable costs that involve screening yield and productive use of the BHC. We will also model revenue sources and the ability to use codes which allow for billing of the screening. From this model, we will measure outcomes such as time to break-even costs of investing in SBIRT and return on investment in SBIRT for each of the sites included in this study.

### Data analysis

A total of 600 patients were randomized to the BI/RT or BI/RT+ conditions, and we stratified based on site, behavioral health consultant, and drug or alcohol severity (mild, moderate, or severe based on AUDIT or DAST-10 score). These stratification variables were chosen to account for possible differences in intervention delivery and treatment response. Specifically, we aimed to account for general site differences such as neighborhood/population served, typical amount of clinical contact received by patients, and range of services available. Similarly, we chose to stratify randomization by BHC in order to equalize non-specific provider variables across conditions. Because severity of substance use has consistently been demonstrated to moderate the impact of brief intervention on substance use [13], we equalized the proportion of mild, moderate, and severe substance use across conditions. Prior to performing all analyses, standard data screening/cleaning procedures will be applied. These procedures will (1) re-screen the data for data-entry errors, (2) check for outliers, (3) assess the extent and pattern of missing data, and (4) check that appropriate assumptions of normality are met and employ remedial measures such as power transformations whenever necessary. The randomization will be checked by comparing the groups on relevant background variables and on all baseline dependent measures. The comparisons will use analyses of variance for continuous variables and log-linear models for discrete or ordinal responses. Some covariates will be considered for inclusion in the main analyses to improve the precision of our hypothesis testing [56]. In addition, we have powered the study sufficiently to conduct subgroup analyses. Based on the literature, it is possible that patients who are using harder illicit drugs (cocaine, heroin, etc.) may respond differently to BI/RT and BI/RT+ than patients who are only using marijuana, or who use marijuana but primarily have a problem with alcohol. We will be adequately powered to break out these subgroups for secondary analysis. Below we specify each hypothesis and discuss the statistical procedures we will employ in analyzing the data.

#### **Primary H1**

Patients assigned to BI/RT+ will attend more substance intervention and community-based specialty treatment sessions outside of the FQHC over the 12-month follow-up than patients assigned to BI/RT.

A linear mixed effects model [57] will be used to compare participants in the two groups on the number of sessions attended in the past 90 days at the 3-, 6-, 9-, and 12-months follow-up assessments. The model will include terms for intervention group, drug type (i.e., illicit drug or alcohol/marijuana), time, BHC/site, and relevant interactions along with any necessary covariates. For these analyses we will combine alcohol and marijuana use given the high prevalence of co-occurring use as well as the social acceptability and decriminalized status of marijuana in Philadelphia, which can contribute to a decreased perception for need for treatment. Specific contrasts will compare (1) differences between individuals in the illicit drug sub-group who received the BI/RT intervention and those who received the BI/RT+ intervention and (2) differences between individuals in the alcohol/marijuana sub-group who received the BI/RT intervention and those who received the BI/RT+ intervention. The mixed effects model will be performed using SAS’s PROC MIXED.

#### **Primary H2**

Patients assigned to BI/RT+ will demonstrate larger reductions in drug use by point prevalence urine samples and by reported days using over the 12-month follow-up compared to patients in BI/RT.

A generalized estimating equations (GEE) model [58] will be used to compare participants in the two groups on rate of urine-confirmed drug abstinence at each follow-up assessment, and linear mixed effects models will be used to examine days of self-reported drug use in the past 30 days at each follow-up assessment. The models will be similar to those outlined for H1, and the baseline response variable (i.e., urine result or days drug use) will be entered as a covariate in the model. As with H1, contrasts will examine the effects of the intervention separately for the two drug type sub-groups. Analyses will be performed using SAS’s PROC GENMOD (GEE) and PROC MIXED.

#### **Primary H3**

BI/RT+ will have positive net social benefits relative to BI/RT alone.

As described above, we will measure social benefits (e.g., downstream medical costs, reduced crime) and costs related to the interventions. We will then test whether marginal costs, marginal benefits, and net benefits of the interventions are different than zero.

#### **Secondary H4**

Patients assigned to BI/RT+ will demonstrate better medical, employment, legal, and psychiatric functioning, as well as reduced HIV risk over the 12-month follow-up compared to patients in BI/RT.

Linear mixed effects models will be used to compare participants in the two groups on measures of psychosocial functioning as assessed by the standard summary scores of the ASI (Medical, Employment, Legal, and Psychiatric) at each follow-up assessment. We will also calculate an overall HIV risk score as assessed by the HIV-RA at each follow-up point. The models will be similar to those outlined for H1, and the baseline response variable will be entered as a covariate in the model. As with H1, contrasts will examine the effects of the intervention separately for the two drug type sub-groups.

### Power for the primary hypotheses

Power analyses are based on the sub-group contrasts identified for each of the primary hypotheses. The analyses are based on a two sided alpha of .0125 (.05/4 outcome variables–number of treatment sessions attended, urinalysis-confirmed abstinence, days of self-reported drug use, and cost of acute care), an estimated correlation of .5 between the repeated measurements where applicable, and a 20 % attrition rate by month 12. Power calculations were based on Diggle, Heagerty, Liang, and Zeger [58] for the mixed effects models, Hedeker, Gibbons, and Waternaux [59] for the GEE models, and Cohen [60] for the cost-related hypotheses. With a sample size of 100 per condition in the illicit drug sub-group, we will have 80 % power to detect moderate effects of the intervention (*d* = .5) for the mixed effects models and cost-related analysis and 80 % power to detect a 20 % difference between the two groups for the GEE model. With a sample size of 200 per condition in the alcohol/marijuana sub-group, we will have 80 % power to detect smaller effects of the intervention (*d* = .25–.30) for the mixed effects models and cost-related analysis and 80 % power to detect smaller differences (13–15 %) between the two groups for the GEE model.

## Discussion

The research described here is designed to make important contributions to the substance abuse treatment field and will address practical scientific questions that could have direct bearing on the use of SBIRT in of primary care practices. We will examine whether an expanded brief intervention in a primary care setting has additional value over traditional SBIRT alone.

The expanded BI/RT+ intervention is promising and may lead to better results because it is designed to help patients resolve ambivalence about engaging in more healthy behaviors, and then support patients as they commit to plans to change. Many patients, particularly those with more problematic use, may not initially feel ready to engage in action steps towards changing their substance use. These patients are likely to benefit from an enhanced, multi-session brief intervention.

The BI/RT+ intervention integrates several innovative features. First, BI/RT+ involves a full 2–6 session MET intervention as opposed to one MI session in traditional SBIRT. The advantage of MET over a single session of MI as a brief intervention is the integration of feedback, the opportunity for patients to try a change strategy and adjust it if it is not working, and to involve significant others in key sessions to shore up motivation and decisions to change [61]. A single session brief intervention may be quite galvanizing towards change and action, but may not be enough to sustain change for particularly problematic substance use. Illicit substance users, particularly those with moderate or severe use, may need more support when preparing to change, and to maintain and follow-through on the decision to enter treatment. Second, BHCs using the BI/RT+ intervention will have a menu of evidence-based options to help patients. The health education toolkit designed for this study provides clinicians with materials that allow them to incorporate various counseling strategies that have been demonstrated to be effective for patients with SUDs, including CBT, MET, and 12-step Facilitation. BHCs can use the toolkit materials to tailor their counseling sessions to meet the patient’s individual needs. Finally, in our enhancements to SBIRT, BHCs engage patients in ongoing telephone monitoring monthly after the patient has completed face-to-face interventions. As addiction has increasingly been conceptualized as a chronic disease [62], the concept of conducting ongoing recovery monitoring and intervening in the case of deterioration has been proposed [63].

Given the mixed results in the current SBIRT literature, this study will also further our understanding of SBIRT’s effectiveness. The results of this study will help shed light on when SBIRT works, for whom, and what intensity is most appropriate for various substances and severities. For example, we may find that BI/RT works well for patients using some substances, but patients using other substances may respond better to the BI/RT+ level of care. Our subgroup analyses will examine whether patients who are using harder illicit drugs (cocaine, heroin, etc.) respond differently to BI/RT and BI/RT+ than patients who are primarily using marijuana or alcohol. We will examine a range of outcomes that are important to patients including substance use, engagement in treatment, and medical and psychosocial functioning. These results will further our understanding of SBIRT’s applications and limitations. Additionally, a strength of this study is that it was conducted in FQHCs with a primarily low-income, Black/African American patient population. Thus this study will provide important information about the effectiveness of these interventions for a traditionally underserved population.

While this study will advance our knowledge about SBIRT, there are a few important limitations to note. First, we did not include a no-treatment control condition where patients receive screening only with no brief intervention. A no-treatment control condition would demonstrate the comparative effectiveness of BI/RT and BI/RT+ and provide a more certain estimate of the true effectiveness of the two brief interventions. We chose not to include a no-treatment condition for ethical reasons. We consider it unethical to identify a patient’s substance use problem and then not provide any services to address the problem. However, this research will enable our team to answer the question of whether providing a BHC the latitude to conduct expanded brief intervention (BI/RT+) at the primary care location yields significantly improved patient outcomes.

A second limitation is the potential for condition contamination. In this study, each BHC provided both BI/RT and BI/RT+. The alternate method would be to assign independent BHCs to each condition. The primary advantage associated with nesting providers across interventions, such that each BHC provides both interventions, is that non-specific provider variables are equalized across conditions. The disadvantage associated with this approach is the increased likelihood of condition contamination or “bleeding,” given that providers are administering both interventions and may introduce methods of one intervention into the other. We have elected to nest BHCs across conditions because: (1) we can identify, specify, and measure “bleeding” more clearly than non-specific provider variables; (2) we can take actions to manage protocol violations; and (3) we can assess the influence of intervention integrity on our dependent measures more readily than we can assess the influence of non-specific provider factors.

A final limitation is that we did not conduct hair analysis and used only urinalysis. Hair testing would have allowed us to obtain a biological measure of substance use covering a longer period of time.

By conducting a large-scale randomized trial in real-world health centers, this study will answer important questions about the effectiveness of expanded SBIRT in primary care settings. Furthermore, our economic evaluation and process evaluation will provide additional context for health systems that are considering implementing brief treatment interventions.
